# A Case of Complete Dislocation of the Outer Extremity of the Clavicle

**Published:** 1915-12

**Authors:** Edwin L. Bebee

**Affiliations:** Buffalo


### A Case of Complete Dislocation of the Outer Extremity of the Clavicle.

DR. EDWIN L. BEBEE, Buffalo.

P. S., a chauffeur, was injured when the heavy touring car which he was driving capsized. He was thrown out on his left side. The edge of the body came down on the right shoulder, striking just below the accromian process, on the deltoid overlying the head of the humerus. The humerus with the scapula was forced downward, backward and inward. The outer end of the clavicle was forced upward over the acromian process. All the ligaments uniting the clavicle to the scapula were torn. Not only the superior and inferior acromio-clavicular, but also the conoid and the trapezoid.

He complained of pain in the shoulder, and inability to use the arm, or to move it without pain. Inability to raise the arm. The right shoulder was lower than the left. The outer extremity of the clavicle was elevated to make a marked prominence, whose outlines were perfectly distinct.

Reduction was accomplished without difficulty. The surgeon's hand in the axilla, the patient's arm by his side, traction outward was made while the end of the clavicle was pressed into place.

An attempt was made to maintain the reduction by Stimpson's apparatus. A strip of adhesive, three and one-half inches wide, was carried under the elbow, and upward in front of and behind the arm, to cross above over the outer end of the clavicle, and extend down and inward beyond the point of crossing, about four inches. This was reinforced by a muslin bandage passed in alternate turns around the body; and from under the elbow over the shoulder, of the affected side. This bandage was fixed in position by sewing the turns together.

Such a dressing would have been sufficient to hold the bones in place in case of incomplete dislocation. When the coraco-clavicular ligaments being intact the tendency to displacement is very much less. In this case all was well as long as the patient remained quiet on his back. But when he moved about or resumed the upright position, the dislocation soon recurred. Therefore an operation was decided upon.

Under ether a four inch incision was made transversely over the joint. The joint was cleared out and the dislocation reduced. A point half an inch behind the articular surface in each bone was drilled straight through, from without inward away from the skin surface. Here the margins of the acromian and the clavicle approach one another at an acute angle. So that a suture can be introduced without invading the joint, which will hold the bones in place. A copper wire of rather large gage was selected. This was carried through the hole in the clavicle, from without in, in the direction of drilling. The end was brought out between the bones, and carried through the acromian in the same direction. It was then brought out between the bones to meet the other end. Thus a figure of eight was formed, which effectually prevented the bones from overriding one another. It was planned to remove this suture later if it proved a source of irritation.

There was no recurrence of the deformity. The wound healed promptly. Motion was allowed after three weeks. The patient soon resumed his occupation with complete use of the shoulder. There was no pain or limitation of motion. There was some tenderness or palpation over the suture at the points where it entered the bone. But evidently the irritation has been negligable as the patient has not applied for its removal.

An absorbable suture of chromic gut or kangaroo tendon would have been preferred, but it was feared that on account of the man being large and muscular the suture would not hold until union of the ligaments was firm. For this same reason copper wire of rather large size was selected instead of silver wire.

It is usually recommended to drill obliquely, so that the ligature will pass from the outer surface of the bone through the articular surface. This method requires wider exposure. Is more difficult. The ligature tends to break out, on account of the thinness of the bone. When drilling through the whole thickness one can easily take as big a bite as is necessary. It is less likely to heal kindly on account of the access of the synovial fluid. It is more apt to give an immobile and tender joint.

---

**Influence of War on Prostitution.** Butte, Bull. et Mem. de la Soc. de Med. de Paris, Sept. 24. For the year ending July 31, 1914, 14,851 visits were made to girls in houses, 2 syphilitic and 2 non-syphilitic venereal cases being found. For the year ending July 31, 1915, the numbers were 8,348 0 and 2. Regular visits to girls not in houses, 1913-14, 48,071, 32 syphilitics and 47 non-syphilitics; 1914-15, 26,174, 22 and 65. Street walkers and amateurs arrested, 1913-14, 46,228, 158 syphilitics, 326 non-syphilitics; 1914-15, 45,452, 209 and 871. Unregistered girls, 1913-14, 3,201, 142 syphilitics, 644 non-syphilitics; 1914-15, 3,907, 167 syphilitics, 939 non-syphilitics.

